# First‐line *nab*‐paclitaxel plus carboplatin for patients with advanced non‐small cell lung cancer: Results of the NEPTUN study

**DOI:** 10.1002/cam4.4310

**Published:** 2021-10-20

**Authors:** Tobias Dechow, Jorge Riera‐Knorrenschild, Björn Hackanson, Jan Janssen, Holger Schulz, Marco Chiabudini, Ludwig Fischer von Weikersthal, Stephan Budweiser, Axel Nacke, Dagmar Taeuscher, Manfred Welslau, Karin Potthoff

**Affiliations:** ^1^ Practice for Oncology Ravensburg Ravensburg Germany; ^2^ University Hospital Giessen and Marburg GmbH Marburg Germany; ^3^ University Medical Center Augsburg Augsburg Germany; ^4^ Practice for Hematology and Oncology Westerstede Germany; ^5^ Practice for Internal Oncology and Hematology Frechen Germany; ^6^ Department of Biostatistics iOMEDICO Freiburg Germany; ^7^ Health Center St. Marien GmbH Amberg Germany; ^8^ Hospital Rosenheim Rosenheim Germany; ^9^ Practice for Hematology and Oncology Remagen Germany; ^10^ SRH Wald‐Klinikum Gera GmbH Gera Germany; ^11^ Medical Care Center at Hospital Aschaffenburg GmbH Aschaffenburg Germany; ^12^ Medical Department iOMEDICO Freiburg Germany

**Keywords:** carboplatin, Germany, *nab*‐paclitaxel, non‐small cell lung carcinoma (NSCLC), real‐world

## Abstract

**Background:**

Platinum‐based chemotherapy remains a first‐line standard of care for approximately 30% of patients with non‐small cell lung cancer (NSCLC) not harboring a druggable alteration. Favorable efficacy and safety of the *nab*‐paclitaxel/carboplatin (*nab*‐P/C) combination was shown in the pivotal phase 3 trial. However, information on effectiveness of *nab*‐P/C in a real‐world setting in Germany is missing. The NEPTUN study prospectively investigated the effectiveness and safety of *nab*‐P/C in patients with advanced NSCLC in a real‐world setting.

**Methods:**

Patients with advanced or metastatic NSCLC received first‐line *nab*‐P/C according to clinical routine. The primary endpoint was 6‐month progression‐free survival rate (PFS6). Other endpoints included further effectiveness parameters, safety and quality of life. Data were analyzed descriptively.

**Results:**

408 patients were enrolled. PFS6 was 40.8% (95% confidence interval [CI], 35.3–46.2); median PFS was 5.2 months (95% CI, 4.5–5.7). overall response rate was 41.5% (95% CI, 36.3–46.8). Median overall survival (OS) was 10.5 months (95% CI, 9.2–11.6). Subgroup analyses revealed median OS for squamous versus non‐squamous histology (11.8 months [95% CI, 9.2–13.8] vs. 9.6 months [95% CI, 7.7–11.2]) and age ≥70 versus <70 years (11.7 months [95% CI, 9.4–14.3] vs. 9.6 months [95% CI, 7.5–11.2]). Most common treatment‐emergent adverse events (TEAEs) were anemia (26.5%), leukopenia (25.7%), and thrombocytopenia (16.6%). Mostly reported grade 3/4 TEAEs were leukopenia (10.2%), anemia (8.6%), and pneumonia (5.1%). *nab*‐paclitaxel‐related deaths as reported by the investigator occurred in 0.8% of patients.

**Conclusion:**

These real‐world data support the effectiveness and safety of *nab*‐P/C as first‐line treatment for patients with advanced NSCLC independent of tumor histology. The results are comparable with the pivotal phase 3 trial. No new safety signals emerged.

## Graphical abstract

The NEPTUN noninterventional study evaluated the effectiveness and safety of *nab*‐paclitaxel and carboplatin as first‐line therapy in patients with advanced or metastatic non‐small cell lung cancer (NSCLC). The results of the NEPTUN study underline that this combination regimen is active and well tolerated in patients with NSCLC and squamous as well as nonsquamous histology and, thus, support the use of *nab*‐paclitaxel and carboplatin as first‐line therapy independent of histology. No new safety signals emerged.

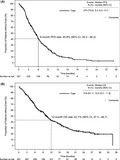

## 1 INTRODUCTION

Non‐small cell lung cancer (NSCLC) is one of the most common malignancies and the leading cause of cancer death among both men and women worldwide.^1^, ^2^ Regarding histological classification, adenocarcinoma and squamous cell carcinoma account for the majority of cases. Adenocarcinoma is the most common form of lung cancer, accounting for about 40% of all NSCLC cases. It is the most common histologic subtype in never‐smokers and females. Squamous cell carcinoma presents the second most common NSCLC subtype and is strongly associated with tobacco smoking.

Surgery remains the key treatment option for the treatment of early‐stage NSCLC, accompanied by various systemic therapies and radiation. Nevertheless, the 5‐year survival rate is very poor. Systemic therapy for advanced NSCLC usually consists of chemotherapy, targeted therapy or immunotherapy, or a combination of these.^3^ Tyrosine kinase inhibitors have markedly improved treatment options for patients with genetic aberrations such as epidermal growth factor (EGFR) and anaplastic lymphoma kinase (ALKs). But only a small fraction of patients currently benefits from targeted therapies like EGFR‐, ALK‐, ROS1‐, or BRAF‐inhibitors due to the relatively low mutation frequency.^4^, ^5^ About one‐third of the NSCLC patients without targetable alteration show high programmed cell death‐ligand 1 (PD‐L1) expression ≥50% and therefore qualify for pembrolizumab monotherapy^6^; the remaining majority is thus frequently treated with chemotherapy‐based therapy regimens. According to current guidelines of the American Society of Clinical Oncology (ASCO) and the European Society for Medical Oncology (ESMO) the *nab*‐paclitaxel plus carboplatin combination (*nab*‐P/C) is a recommended standard first‐line treatment regimen for patients with advanced NSCLC without druggable alteration.^7^, ^8^, ^9^

In recent years, immunotherapy was approved as first‐line therapy either as monotherapy for NSCLC patients with PD‐L1 expression ≥50% or in combination with different chemotherapy backbones independent of PD‐L1 expression based on pivotal phase 3 trials Keynote‐024, Keynote‐189, Keynote‐407, IMpower150, IMpower133. Efficacy of immunotherapy (‐combinations) proved to be superior to sole chemotherapy regimens^6^, ^10^, ^11^, ^12^, ^13^ and lead to an improved quality of life (QoL).^14^, ^15^ Some of these immunotherapy regimens make use of the *nab*‐P/C combination as chemotherapy backbone.^11^, ^13^ For example, the KEYNOTE‐407 trial used pembrolizumab in combination with *nab*‐P/C.^11^ In addition, patients with specific comorbidities can benefit from treatment with *nab*‐P/C, as for example efficacy and safety in patients with interstitial lung disease has recently been reported.^16^, ^17^

*nab*‐P/C was approved for first‐line treatment of advanced NSCLC in 2012. The pivotal phase 3 trial compared solvent‐based paclitaxel with a 130‐nm albumin‐bound paclitaxel (*nab*‐paclitaxel) formulation, both in combination with carboplatin. The *nab*‐paclitaxel combination showed an increased overall response rate (ORR) mainly driven by patients with squamous NSCLC. In addition, patients suffered significantly less from neuropathy and arthralgia grade 3 and 4 when treated with *nab*‐paclitaxel compared to those treated with solvent‐based paclitaxel.^18^ These results were confirmed in the consecutive ABOUND studies where elderly patients and patients with poor performance status were treated with *nab*‐P/C.^19^, ^20^ But real‐world data for the *nab*‐P/C combination regimen are scarce.

Aim of the NEPTUN study was to prospectively investigate the effectiveness and safety of *nab*‐P/C in patients with advanced NSCLC in a real‐world setting.

## 2 PATIENTS AND METHODS

### 2.1 Study design and patient eligibility

NEPTUN was a prospective, single‐arm, multicenter, noninterventional study in Germany. The study was designed to evaluate the effectiveness, tolerability and safety, and patient‐reported outcomes (PROs) on QoL of *nab*‐P/C in a real‐world setting. The trial has been approved by the responsible ethics committee and is registered at clinicaltrials.gov (NCT02799862). Before start of individual documentation, eligible patients provided written informed consent.

Eligible patients were ≥18 years old, had histologically confirmed locally advanced or metastatic NSCLC, and were not candidates for curative treatment. Patients received no previous systemic therapy for advanced disease and have been assigned to or have received *nab*‐P/C combination therapy not more than 4 weeks before providing informed consent.

### 2.2 Treatment and study procedures

Patients received study medication according to clinical routine and current German *nab*‐paclitaxel summary of product characteristic (SmPC; the decision for *nab*‐P/C prescription was clearly separated from and prior to the decision to include patients into the study). According to current SmPC, *nab*‐paclitaxel was administered as 100 mg/m² infusion on day 1, 8, and 15 of each 21‐day cycle, and carboplatin was administered as area under the curve 6 mg · min/ml infusion on day 1 of each 21‐day cycle. All assessments and procedures were performed as per local clinical practice.

Tumor responses were assessed by the investigator according to local standards and clinical routine.

Adverse events (AEs) were reported until 30 days after the last dose of *nab*‐paclitaxel.

Patient‐reported outcomes on QoL were assessed at baseline before start of therapy, week 6, week 12, and every 3 months thereafter until progressive disease (PD) or start of next anticancer therapy by using the self‐report instruments Functional Assessment of Cancer Therapy‐Lung (FACT‐L) and EuroQoL 5‐Dimension 5‐Level (EQ‐5D‐5L). Additionally, patients were asked to report PROs on QoL at end of treatment visit and at PD. Patients had to provide separate consent for participation at the questionnaire project.

### 2.3 Objectives and endpoints

Primary objective was to evaluate effectiveness of the *nab*‐P/C combination therapy in a real‐world setting. Primary endpoint was the 6‐month progression‐free survival rate (PFS6).

Secondary objectives were to obtain further data on effectiveness measured by progression‐free survival (PFS), overall survival (OS), ORR, and disease control rate (DCR). Additionally, safety and tolerability and PROs on QoL were assessed.

The long‐term follow‐up of the NEPTUN study was finished in June 2021. The analysis presented here is a pre‐planned interim analysis that includes the final analysis of the primary endpoint. Data cutoff for this analysis was 7 December 2019.

### 2.4 Statistical analyses

For this study, no formal hypotheses were given. Statistical analyses are explorative in nature. All parameters were analyzed descriptively. Time‐to‐event analysis variables (PFS and OS) and their fixed‐time estimators (PFS6 and 12‐month OS rate [OS12]) were analyzed using the Kaplan–Meier method. PFS was defined as the time from the start of therapy to the date of PD or death from any cause. OS was defined as the time from the start of therapy to the date of death from any cause. For PFS analysis, patients that have not experienced an event were censored with the date of last contact or with begin of the following antineoplastic therapy, whichever comes first. If death was not reported by the time of analysis cutoff, OS was censored at with the date of last contact. For PFS, a Cox proportional hazard model was used to estimate hazard ratios (HRs) for the potentially prognostic covariates (Eastern Cooperative Oncology Group [ECOG] performance status [0/1 vs. ≥2], histology [squamous vs. nonsquamous], age group [≥70 vs. <70], renal impairment based on creatinine clearance [normal—CrCl ≥90 ml/min; mild—CrCl ≥60 to <90 ml/min; moderate—CrCl ≥30 to <60 ml/min; severe—CrCl <30 ml/min], and smoking status [never‐smoker, former smoker, current smoker]). ORR was defined as proportion of patients achieving complete response (CR) or partial response (PR) as best response. DCR was defined as proportion of CR, PR or stable disease as best response. Safety and tolerability were assessed in all patients that received at least one dose of study therapy according to SmPC. AEs were coded using MedDRA version 21.0 and severity was graded according to Common Terminology Criteria for Adverse Events (CTCAE) version 4.03.^21^ The relative dose intensity was calculated relative to the recommended dose according to SmPC. Treatment duration was calculated using the Kaplan–Meier method. Patients with ongoing therapy by the time of analysis were censored with their last application date. Scales, subscales, and single items of the QoL questionnaires EQ‐5D‐5L and FACT‐L were calculated according to the respective manuals.

## 3 RESULTS

### 3.1 Patients

**FIGURE 1:**
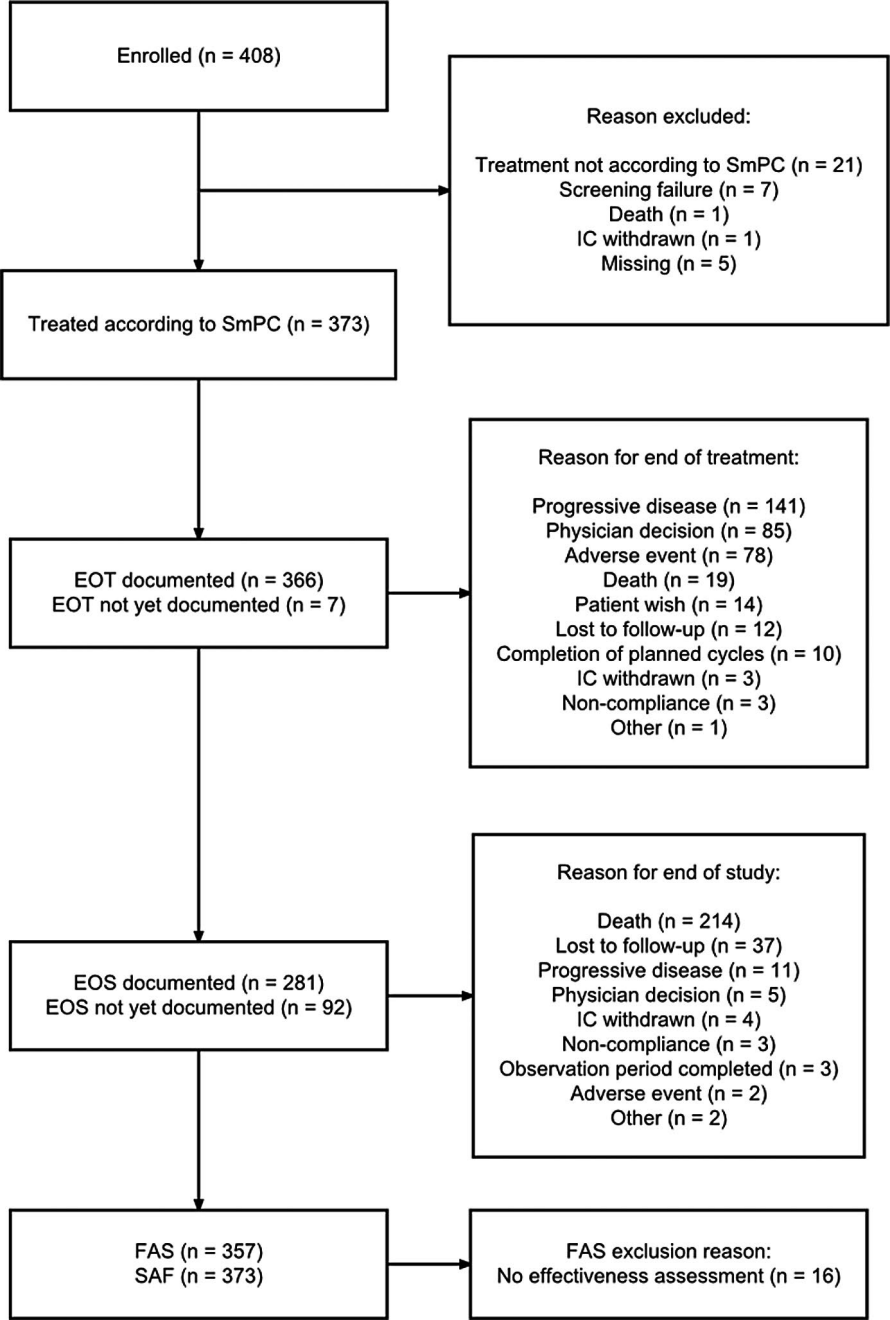
Patient disposition. Patients with either treatment start or date of informed consent prior to 6 months before database cut were included into this analysis. EOS, end of study; EOT, end of treatment; FAS, full analysis set; IC, informed consent; SAF, safety analysis set; SmPC, summary of product characteristics

Between August 2016 and June 2019, 408 patients had been enrolled at 75 oncological hospitals and practices. Of those, 373 patients were treated according to the SmPC and were evaluated for safety (safety analysis set); 357 patients had at least one effectiveness assessment and thus qualified for effectiveness analysis (full analysis set). Disposition of patients is shown in Figure 1 (CONSORT flow diagram).

Median age of patients was 67.7 years (min 44, max 87 years) and the majority (63.3%) was younger than 70 years. Patients were predominantly male (70.6%), current or former smokers (79.6%), and presented with an ECOG performance status ≤1 (71.1%). Adenocarcinoma was the most common histology (49.3%) followed by squamous cell carcinoma (40.1%). A total of 82.9% of patients presented with distant metastases at baseline, with bones and distal lymph nodes most frequently affected (25.2% each), followed by liver (17.9%) and brain (15.7%). Further patient baseline characteristics and demographics are summarized in Table 1.

**TABLE 1:** Baseline demographic and clinical characteristics

Characteristic	All patients (*n* = 357)	
	*n*	%
Age, years		
Median	67.6	
Range	44–87	
<70	226	63.3
≥70	131	36.7
Sex		
Male	252	70.6
Female	105	29.4
ECOG performance status, *n* (%)		
0	88	24.6
1	166	46.5
2	64	17.9
3	8	2.2
Missing	31	8.7
Histology		
Adenocarcinoma	176	49.3
Squamous cell carcinoma	143	40.1
Large cell carcinoma	20	5.6
Other	17	4.8
Missing	1	0.3
PD‐L1 status		
PD‐L1 positive	85	23.8
PD‐L1 negative	107	30.0
No PD‐L1 status available	165	46.2
Distant metastases at baseline		
Yes	296	82.9
No	60	16.8
Missing	1	0.3
Prior antineoplastic therapy		
Radiotherapy	102	28.6
Chemotherapy	20	5.6
Smoking status		
Never smoked	71	19.9
Smoked and quit	173	48.5
Smoked and still smokes	111	31.1
Missing	2	0.6

Abbreviations: ECOG, Eastern Cooperative Oncology Group; PD‐L1, programmed cell death‐ligand 1.

### 3.2 Effectiveness

#### 3.2.1 Progression‐free survival

**FIGURE 2:**
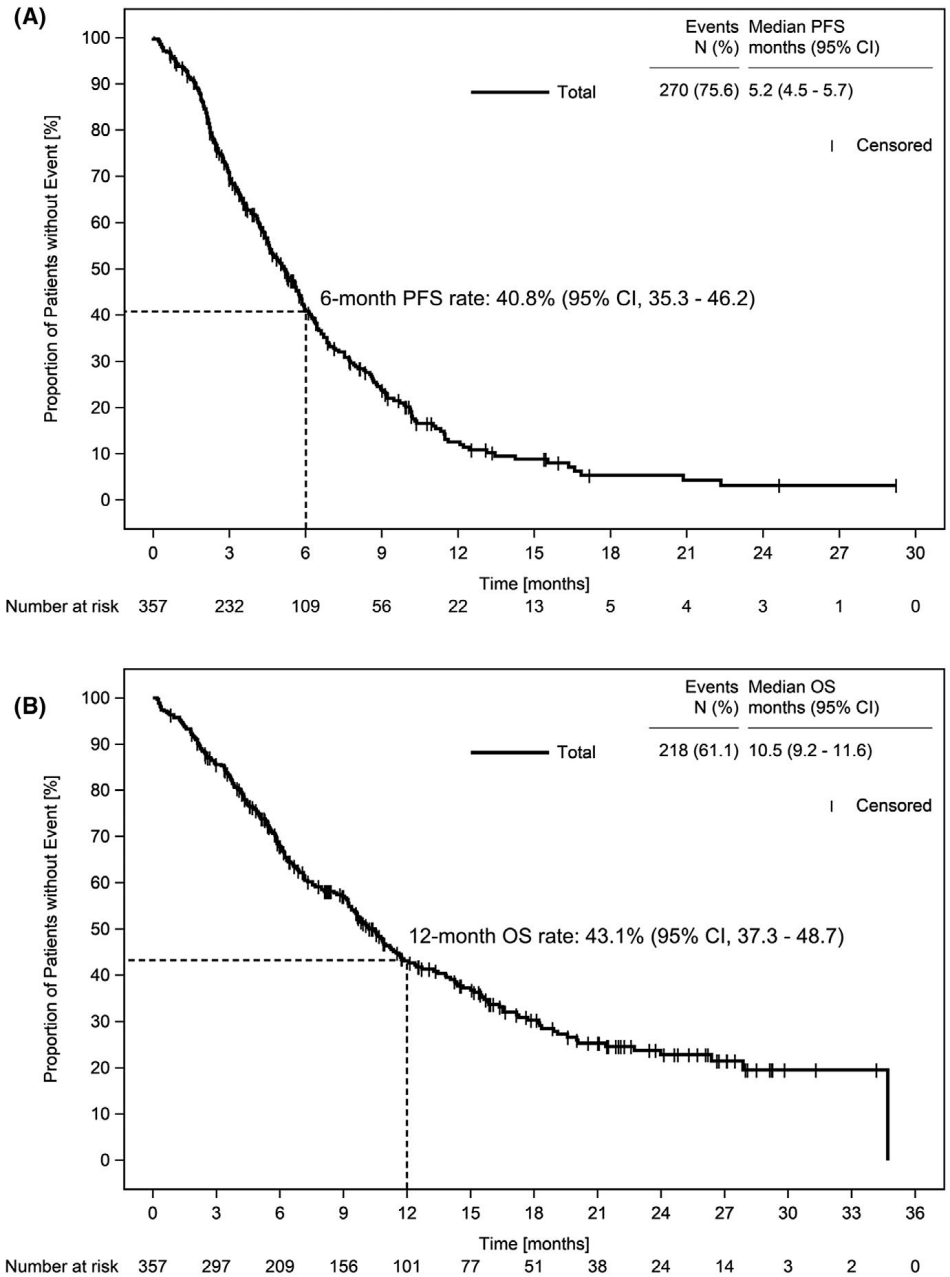
Effectiveness. PFS and OS by Kaplan–Meier estimates of 357 patients included in the full analysis set; PFS and PFS6 (A), OS and OS12 (B). OS, overall survival; OS12, 12‐month OS rate; PFS, progression‐free survival; PFS6, 6‐months PFS rate

PFS6 was 40.8% (95% confidence interval [CI], 35.3–46.2), and median PFS was 5.2 months (95% CI, 4.5–5.7) (Figure 2A). Elderly patients aged ≥70 years had a median PFS of 5.8 months (95% CI, 4.9–6.8) compared to 4.6 months (95% CI, 3.9–5.3) for patients <70 years of age. For patients with squamous cell histology, a median PFS of 5.6 months (95% CI, 4.6–6.3) compared to 4.6 months (95% CI, 4.1–5.6) for patients with non‐squamous histology was reported. A multivariable cox regression model showed that squamous histology and age ≥70 years tend to result in a reduced risk for a PFS event (HR for squamous vs. non‐squamous histology 0.76 [95% CI, 0.58–1.01]; HR for age ≥70 years vs. <70 years of age: 0.80 [95% CI, 0.59–1.08]).

#### 3.2.2 Overall survival

Median OS was 10.5 months (95% CI, 9.2–11.6); OS12 was 43.1% (95% CI, 37.3–48.7) (Figure 2B). Median OS for elderly patients ≥70 years was 2.1 months longer compared with patients <70 years (11.7 months [95% CI, 9.4–14.3] vs. 9.6 months [95% CI, 7.5–11.2]), and patients with squamous histology survived 2.2 months longer compared to patients with nonsquamous histology (median OS: 11.8 months [95% CI, 9.2–13.8] vs. 9.6 months [95% CI, 7.7–11.2]).

#### 3.2.3 Response

Based on investigator‐assessed response evaluation according to local routine, ORR and DCR were 41.5% (95% CI, 36.3–46.8) and 61.6% (95% CI, 56.4–66.7), respectively. Specifically, 1.7% of patients achieved a CR and 39.8% a PR. Patients with squamous histology responded slightly better to *nab*‐P/C compared to patients with nonsquamous histology. ORR for patients with squamous histology was 45.5% (95% CI, 37.1–54.0) and 38.5% (95% CI, 31.9–45.4) for patients with nonsquamous histology (Table 2).

**TABLE 2:** Response rates for the FAS population and histologic subsets

	FAS (*n* = 357)		Nonsquamous (*n* = 213)		Squamous (*n* = 143)	
	*n*	% (95% CI)	*n*	% (95% CI)	*n*	% (95% CI)
Best response						
Complete response	6	1.7	3	1.4	3	2.1
Partial response	142	39.8	79	37.1	62	43.4
Stable disease	72	20.2	43	20.2	29	20.3
Progressive disease	83	23.2	60	28.2	23	16.1
Not evaluable	2	0.6	1	0.5	1	0.7
No tumor assessment available	52	14.6	27	12.7	25	17.5
Overall response rate	148	41.5 (36.3–46.8)	82	38.5 (31.9–45.4)	65	45.5 (37.1–54.0)
Disease control rate	220	61.6 (56.4–66.7)	125	58.7 (51.8–65.4)	94	65.7 (57.3–73.5)

Note

One patient was excluded due to a missing value for histology.

Abbreviations: CI, confidence interval; FAS, full analysis set.

### 3.3 Treatment details

During study participation, patients were treated according to clinical routine and current German SmPC. Median treatment duration was 3.2 months (95% CI, 3.0–3.6) for *nab*‐paclitaxel and 3.4 months (95% CI, 3.0–3.7) for carboplatin. Mean relative dose intensity was 74.3% (min 16.0%, max 104%) for *nab*‐paclitaxel and 75.1% (min 21%, max 102%) for carboplatin. *nab*‐paclitaxel dose interruptions were reported for 82.6% of the patients while carboplatin dose interruptions were observed less frequently (62.7%). AEs were reported most often as reason for dose interruptions (*nab*‐paclitaxel: 63.5%; carboplatin: 35.9%) followed by organizational reasons (*nab*‐paclitaxel: 47.5%; carboplatin 39.5%). Dose reductions and dose withdrawals for *nab*‐paclitaxel occurred in 31.9% and 27.6% of patients, respectively. For carboplatin, dose reductions were reported in 56.6% and dose withdrawals in 5.6% of the patients.

### 3.4 Safety

Treatment‐emergent adverse events (TEAEs) of any grade were reported in 341 of 373 patients (91.4%), with anemia and leukopenia as the most common (reported in 26.5% and 25.7% of patients, respectively; Table 3). Grade 3/4 TEAEs were documented in 202 patients (54.2%). Most frequent grade 3/4 TEAEs were leukopenia, anemia and pneumonia occurring in 10.2%, 8.6%, and 5.1% of patients, respectively. Fatal TEAEs were reported for 49 patients (13.1%), whereas 29 were attributed to tumor progression. Seven patients died from pneumonia and/or sepsis; for nine patients, deterioration of general condition was given as reason for death and four patients were documented with death due to comorbidity. TEAEs related to *nab*‐paclitaxel of any grade and of grade 3/4 occurred in 242 patients (64.9%) and 105 patients (28.2%), respectively. TEAEs with causal relationship to carboplatin were reported in 218 patients (58.4%), thereof 92 patients with TEAEs grade 3/4 (24.7%). The most common grade 3/4 treatment related TEAEs for *nab*‐paclitaxel as well as carboplatin were anemia (20.9%, 19.8%) and leukopenia (22.3%, 18.8%). Three fatal related TEAEs were reported for both *nab*‐paclitaxel and carboplatin. Related to *nab*‐paclitaxel, three patients died from leukopenia, sepsis and neutropenic sepsis and related to paclitaxel, three patients died from neutropenic sepsis, pulmonary sepsis and sepsis. *nab*‐paclitaxel treatment discontinuation due to a TEAE was documented for 76 patients (20.3%) of which 37 (9.9%) were due to *nab*‐paclitaxel related TEAEs and 28 (7.5%) were due to carboplatin related TEAEs. Most frequently reported TEAEs (reported for ≥5% of patients) and selected TEAEs of interest including severity and treatment relation information are summarized in Table 3.

**TABLE 3:** Most common treatment‐emergent adverse events occurring in ≥5% of patients

Adverse event	All patients (*n* = 373)			
	Any grade *n* (%)	Grade 3/4 *n* (%)	Related to *nab*‐paclitaxel Any grade, *n* (%)	Related to carboplatin Any grade, *n* (%)
Patients with any event	341 (91.4)	202 (54.2)	242 (64.9)	218 (58.4)
Hematological				
Anemia	99 (26.5)	32 (8.6)	78 (20.9)	74 (19.8)
Leukopenia	96 (25.7)	38 (10.2)	83 (22.3)	70 (18.8)
Thrombocytopenia	62 (16.6)	16 (4.3)	48 (12.9)	48 (12.9)
Neutropenia	31 (8.3)	17 (4.6)	23 (6.2)	23 (6.2)
Nonhematological				
Nausea	61 (14.4)	5 (1.3)	44 (11.8)	44 (11.8)
Fatigue	52 (13.9)	5 (1.3)	34 (9.1)	26 (7.0)
Polyneuropathy	42 (11.3)	8 (2.1)	34 (9.1)	10 (2.7)
General physical health deterioration	42 (11.3)	18 (4.8)	16 (4.3)	12 (3.2)
Diarrhea	41 (11.0)	6 (1.6)	24 (6.4)	19 (5.1)
Dyspnea	38 (10.2)	10 (2.7)	1 (0.3)	1 (0.3)
Pneumonia	26 (7.0)	19 (5.1)	7 (1.9)	7 (1.9)

Note

Adverse events were coded using MedDRA version 21.0. Time range: from first administration of *nab*‐paclitaxel until at least 30 days after *nab*‐paclitaxel discontinuation. More than one reported preferred term per patient within a system organ class was possible.

Abbreviation: MedDRA, Medical Dictionary for Regulatory Activities.

### 3.5 Patient‐reported QoL

**FIGURE 3:**
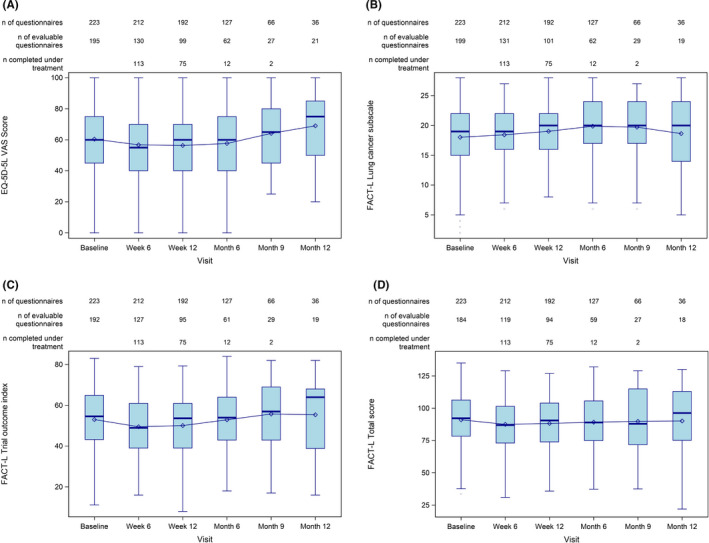
Patient‐reported QoL according to EQ‐5D‐5L VAS Score (A), FACT‐L Lung Cancer Subscale (B), FACT‐L Trial Outcome Index (C), FACT‐L Total Score (D). ‘n completed under treatment’: number of patients who completed the respective item and were still under treatment at the respective time point. EQ‐5D‐5L, EuroQoL 5‐Dimension 5‐Level; FACT‐L, Functional Assessment of Cancer Therapy‐Lung; QoL, quality of life; VAS, visual analogue scale

A total of 223 patients participated in the QoL questionnaire project. FACT‐L and EQ‐5D‐5L questionnaires were answered throughout the observation period. The questionnaire return rate at baseline was 91.5% and decreased with later timepoints to about 50%–60%. EQ‐5D‐5L visual analogue scale score, FACT‐L Lung Cancer Subscale, FACT‐L Trial Outcome Index, and FACT‐L Total Score revealed consistent patient‐reported QoL during treatment (Figure 3).

## 4 DISCUSSION

Real‐world data representative of patients with advanced NSCLC treated with *nab*‐P/C in clinical routine are rare. Several of the previous trials investigating the *nab*‐P/C combination had limitations either due to restricted patient populations^18^, ^19^, ^20^, ^22^ or due to retrospective data collection^23^ and are therefore not representative of the patient population in clinical routine. Therefore, NEPTUN was designed to provide data on effectiveness and safety from routine treatment of an unselected patient population.

The results of this analysis with a median PFS and OS of 5.2 and 10.5 months, respectively, are comparable with those of the pivotal phase 3 trial from 2012 by Socinski et al., showing a median PFS and OS of 6.3 and 12.1 months, respectively.^18^ It must be taken into account that the unselected patient population of NEPTUN includes patients with more comorbidities (>20% of patients had ECOG ≥2), what might result in slightly worse effectiveness results. The ORR in our study was in the same range, but slightly higher compared with Socinski et al. (41.5% vs. 33%). Differences could be partially due to the fact, that in NEPTUN, tumor assessments were done according to local standard and routine clinical practice whereas in the pivotal trial, tumors were assessed by spiral computed tomography scans every 6 weeks until progression. Median treatment duration was shorter in NEPTUN compared to the registration trial (3.2 months vs. 6 cycles [corresponds to 4.5 months]). Dose reductions for *nab*‐paclitaxel were more frequent in the pivotal phase 3 trial compared with dose reductions in NEPTUN (46% vs. 31.9%), whereas dose reductions for carboplatin were more frequent in NEPTUN (56.6% vs. 46%). A shorter treatment duration might be due to an older patient collective with more comorbidities.

Our results revealed that squamous histology and age ≥70 years is associated with a reduced risk for a PFS event and showed extended median OS times of 11.8 and 11.7 months, respectively. Increased survival times in the subgroup of elderly patients were observed earlier in the pivotal trial, revealing a median OS of 19.9 months.^18^ In another study focusing on elderly patients, median OS times of 14.5 months were reached.^20^ A survival benefit for patients with squamous histology could not be confirmed in the existing literature. Socinski et al. rather showed shorter median OS times in patients with squamous histology compared to those presenting with non‐squamous histology (10.7 months vs. 13.1 months).^18^, ^24^ Nevertheless, the NEPTUN results show that patients seem to benefit from *nab*‐P/C treatment independent of histologic subtype.

In choosing an optimal treatment regimen for a patient, QoL is an important factor to consider. In addition to the patient’s well‐being, maintenance of QoL is also important for treatment outcome. As chemotherapy remains the standard of care for the majority of patients with NSCLC and is also used as a backbone for several targeted therapies, QoL data for chemotherapy regimens are an important mean to decide for the best regimen. In NEPTUN, QoL analysis revealed a consistent QoL over the first 12 months of treatment. Published data from Thomas et al. analyzing QoL in patients with squamous NSCLC treated with *nab*‐P/C showed QoL improvements over the first four cycles of treatment.^15^ It would be interesting to see whether this effect last in the maintenance setting of treatment.

Recently, the *nab*‐paclitaxel plus carboplatin combination was used as chemotherapy backbone for several large phase 3 trials investigating PD‐1/PD‐L1 blockade in combination with chemotherapy for therapy of metastatic NSCLC with squamous or non‐squamous histology.^10^, ^11^, ^13^, ^25^, ^26^ Those studies revealed promising results and have been included into the guidelines.^3^ Thus, the addition of pembrolizumab to the chemotherapy regimen resulted in significant improvements in PFS and OS.^11^ Also the addition of atezolizumab to *nab*‐P/C showed significantly improved PFS.^26^ Because combination approaches lead to increasing side effects compared to sole chemotherapy, the choice of the chemotherapy backbone is of utmost importance to reduce toxicity burden for the patient. The results of NEPTUN confirm the known spectrum of side effects as no new or unexpected events occurred. Both the pivotal phase 3 study and the study on elderly patients by Langer et al. showed considerably more grade 3/4 events compared to NEPTUN.^18^, ^20^ This discrepancy might be partially due to an underreporting of AEs that is frequently observed in non‐interventional studies. There might also be a documentation lack since the follow‐up of the NEPTUN study was still ongoing until recently and some patients included in this analysis were still under observation at the time of data cut‐off.

Major strengths of our study are the prospective design and the unselected patient population providing real‐world data on routine treatment of patients with locally advanced or metastatic NSCLC. The fact that our results are comparable with other studies supports the external validity of our data. However, there are also limitations associated with this study and this analysis. As a comparison group is not available due to the single‐arm design and the subgroup analyses used for this work are exploratory in nature, the interpretation and generalizability of results are limited. Results cannot serve as a general decision on the effectiveness of *nab*‐P/C. In the context of other studies in similar patient populations, however, the findings of this study may help to assess how effective and safe *nab*‐P/C is in clinical routine. Since NEPTUN was still ongoing until recently, this analysis contains a relatively high number of missing data, for example regarding response evaluation. As mentioned, safety data are probably prone to underreporting und might still represent unmatured data. With the final NEPTUN analysis, we will present even more mature safety and survival data and will address important patient subgroups, such as patients with liver and brain metastases. Furthermore, non‐stringent methods of assessment for response evaluation due to the real‐world setting have to be considered when interpreting data. In addition, no hypotheses were tested for this analysis and thus only descriptive statistics were performed.

The NEPTUN study provides favorable real‐world outcome, safety and tolerability data of the *nab*‐P/C combination. This frequently used combination remains an important standard treatment option for patients with advanced NSCLC either without or in combination with other substances. Therefore, real‐world data can help in choosing the appropriate therapy strategy for patients treated in daily routine. It will be interesting to monitor which role the *nab*‐P/C will have as treatment option for NSCLC patients in the changing treatment landscape over the upcoming years.

Despite an unselected patient collective including comorbid patients, the combination of *nab*‐P/C proved to be effective and tolerable in the NEPTUN study.

## 5 CONCLUSION

These real‐world data support the effectiveness and safety of *nab*‐paclitaxel plus carboplatin for first‐line treatment of advanced NSCLC independent of tumor histology. The findings are comparable to those reported from the pivotal phase 3 trial. No new safety signals emerged.

## ETHICS STATEMENT

The NEPTUN study was conducted according to the ethical principles of the declaration of Helsinki. The study protocol was reviewed by the independent ethics committee or the institutional review board for each center. Each patient had provided written informed consent before screening procedures were initiated. The trial is registered at ClinicalTrials.gov (NCT02799862).

## CONFLICT OF INTEREST

S. Budweiser, M. Chiabudini, T. Dechow, L. Fischer von Weikersthal, A. Nacke, K. Potthoff, J. Riera‐Knorrenschild, H. Schulz, D. Täuscher, and M. Welslau declare no conflict of interest concerning the topic of this publication. Outside of the submitted work, B. Hackanson received personal fees from Boehringer Inghelheim, MSD, BMS, and Pfizer; J. Janssen received personal fees from Amgen/Onyx, Baxter, Bayer, Biotest, BMS, Celgene, Janssen‐Cilag Ltd, Kedrion Biopharma, Eli Lilly, Merck Serono, Novartis, Octapharma, Pfizer, Pharma Mar, Puma Biotechnology, Roche, Sanofi and Teva Pharmaceutical, Abbvie, Ipsen, and Servier.

## Data Availability

The data that support the findings of this work are available from the corresponding author upon reasonable request.
